# IVMT-Rx-3 Microemulsion as Low-Dose Metronomic Chemotherapy for Melanoma Metastasis

**DOI:** 10.3390/cells15131178

**Published:** 2026-06-29

**Authors:** Rudra Pangeni, Padmanabhan Mannangatti, Ehsan Kaffash, Madeline Gunawardena, Nitai D. Mukhopadhyay, Mark C. Mochel, Swadesh K. Das, Qingguo Xu, Paul B. Fisher

**Affiliations:** 1 Department of Pharmaceutics, Virginia Commonwealth University, Richmond, VA 23298, USAgunawardenam@vcu.edu (M.G.); 2 VCU Institute of Molecular Medicine, School of Medicine, Virginia Commonwealth University, Richmond, VA 23298, USA; 3 Department of Cellular, Molecular and Genetic Medicine, School of Medicine, Virginia Commonwealth University, Richmond, VA 23298, USA; 4 Department of Biostatistics, School of Public Health, Virginia Commonwealth University, Richmond, VA 23298, USA; 5 Department of Pathology, School of Medicine, Virginia Commonwealth University, Richmond, VA 23298, USA; 6 VCU Massey Comprehensive Cancer Center, School of Medicine, Virginia Commonwealth University, Richmond, VA 23298, USA; 7 Department of Ophthalmology, Biomedical Engineering, Pediatrics, Center for Pharmaceutical Engineering, and Center for Drug Discovery, Virginia Commonwealth University, Richmond, VA 23298, USA

**Keywords:** MDA-9/Syntenin, melanoma, PD-L1, metastasis, oral formulation, bioavailability

## Abstract

**Highlights:**

**What are the main findings?**

- Stable, high-drug-loading oral microemulsion (ME) of the MDA-9/Syntenin antagonist IVMT-Rx-3 (IVMT-Rx-3-ME) was developed, enabling improved membrane transport and oral bioavailability compared with the free drug dispersion.
- Oral IVMT-Rx-3-ME suppressed primary tumor growth and lung metastases in murine melanoma models and showed enhanced efficacy in combination with anti-PD-L1, with no observable systemic toxicity.

**What are the implications of the main findings?**

- The oral ME platform improves the translational potential of a potent PDZ-domain antagonist by overcoming poor oral bioavailability and supporting clinically relevant dosing.
- IVMT-Rx-3-ME enables metronomic oral therapy and rational combination with immune checkpoint blockade, providing a promising strategy for aggressive and metastatic melanoma.

**Abstract:**

The pro-metastatic gene MDA-9/Syntenin-1 and its tandem PDZ domains (PDZ1 and PDZ2) provide established targets for intervening in tumor progression and metastasis. Recently, we generated and validated MDA-9/Syntenin-1 antagonists targeting a single PDZ domain (PDZ1i) or both PDZ domains (IVMT-Rx-3) in carcinomas and melanoma. Data reveal that IVMT-Rx-3 possesses immunomodulatory and anti-angiogenic properties, in addition to its well-established anti-invasive capabilities. Despite its significant druggable properties, it cannot be delivered orally, limiting its clinical potential. Here, we characterized an oral microemulsion (ME) formulation of IVMT-Rx-3, IVMT-Rx-3-ME to enhance intestinal permeability, bioavailability, and therapeutic efficacy. Physicochemical analyses demonstrated that the optimized formulation produced a stable IVMT-Rx-3-ME with high drug content (>90%). In vitro permeability and dissolution assays confirmed improved membrane transport and solubility compared with the free drug dispersion control. Pharmacokinetic studies in rats revealed that the ME enabled rapid absorption and sustained systemic exposure, whereas the free drug showed negligible bioavailability. In murine metastatic melanoma models, oral IVMT-Rx-3-ME suppressed tumor growth and lung metastases, and when combined with anti-PD-L1 antibody, produced synergistic antitumor effects with minimal toxicity. Collectively, these findings highlight IVMT-Rx-3-ME as a potent and viable oral metronomic chemotherapy platform for metastatic melanoma, with enhanced combinatorial translational potential with immunotherapies.

## 1. Introduction

Melanoma remains the deadliest form of skin cancer due to its rapid progression, systemic dissemination and resistance to therapy [1]. Conventional (e.g., chemotherapy and radiation), as well as more recent immunotherapies and targeted therapies, offer limited survival benefit in selective subsets of patients, mostly providing palliative roles in advanced metastatic disease. Considering this decimal scenario there is an imperative to develop and employ innovative therapeutic solutions, including identifying novel targets, defining effective molecular interventions and clinically deliverable formulations for successful clinical translation.

Melanoma differentiation associated gene-9 (MDA-9) [2,3], also referred to as Syntenin-1 [4] or Syndecan binding protein, was initially identified as a positive regulator of melanoma metastasis and subsequently established as a significant molecular target in many progressed and malignant cancers [5,6,7]. Metastasis is a complex process, encompassing multiple unique and overlapping steps that are driven by numerous controlling signaling pathways. Accumulated data indicates that most of these steps, e.g., epithelial–mesenchymal transmission [8,9,10,11], cellular migration and invasion [4,12,13], anoikis-resistant growth [14], adhesion [15], immune modulation [16,17], and regulation of immune infiltration in the pre-metastatic niche [18,19], are influenced at multiple levels by MDA-9 and its partner proteins [5]. Consequently, genetic or pharmacological interventions were anticipated to inhibit metastasis, which has been validated by numerous groups independently [5]. IVMT-Rx-3, first published in 2023 [20], is a unique molecule which consists of a small molecule linked to a peptide that blocks both PDZ domains of MDA-9 and has shown enhanced bioactivity in melanoma vs. a single PDZ1 domain inhibitor, PDZ1i [15,18,21,22]. Attaining elevated selectivity and potency is challenging, as numerous inhibitors encounter issues with inadequate distribution, low affinity, or restricted bioavailability in therapeutic applications. While IVMT-Rx-3 has good solubility in PBS, pH 7.4 (4.10 mg/mL) and a reasonable half-life (2.96 ± 1.41 h) following intraperitoneal administration [20], it showed negligible systemic exposure (below LOQ) when delivered orally, which limits applications in the clinic. Thus, despite adequate aqueous solubility under near-neutral conditions, the negligible oral exposure suggests that intestinal absorption is a primary limitation for IVMT-Rx-3. In addition, the dynamic pH environment of the gastrointestinal tract may alter IVMT-Rx-3 solubility and promote drug precipitation, which can further limit absorption. Therefore, we selected a microemulsion (ME) platform to maintain drug solubilization across the gastrointestinal tract while enhancing membrane transport via oil/surfactant-mediated permeation mechanisms.

Microemulsions (MEs) are thermodynamically stable, single-phase colloidal systems that offer multiple pharmaceutical and biopharmaceutical advantages for oral drug delivery [23]. Their high drug-loading capacity and submicron droplet size enhance solubility and facilitate improved intestinal permeation. Additionally, MEs can reduce pH-dependent solubility variability and maintain the drug in a solubilized state throughout the gastrointestinal tract [24]. Moreover, MEs can enhance oral bioavailability by inhibiting intestinal efflux transporters and minimizing the variability in drug absorption caused by food intake [25,26].

Given the favorable attributes of MEs for oral drug delivery, we formulated IVMT-Rx-3 as an oil-in-water (o/w) ME (IVMT-Rx-3-ME) and evaluated various physicochemical properties, e.g., in vitro and ex vivo permeability assessments, and pharmacokinetic profiling in healthy rats. Indeed, preclinical melanoma models show that orally delivered IVMT-Rx-3-ME in combination with anti-PD-L1 regimens significantly improve antitumor effect and control metastases with no host toxicity, suggesting a potential path for successful clinical transition.

## 2. Materials and Methods

### 2.1. Materials

The synthetic scheme for IVMT-Rx-3 synthesis was previously described, and the same preparation lot was used in this study [20]. PD-L1 antibody was purchased from bioXcell (Lenanon, NH, USA). Kolliphor EL, Tween 80, polyethylene glycol 400 (PEG 400), propylene glycol, and all other reagents were purchased from Sigma-Aldrich (St. Louis, MO, USA). Caprylocaproyl macrogol-8-glycerides (Labrasol), diethylene glycol monoethyl pether (Transcutol HP), and propylene glycol monocaprylate (Capryol 90) were provided as a gift by Gattefossé (Saint-Priest, France).

### 2.2. Solid Phase Chemistry and In Vitro Permeability of IVMT-Rx-3 Across Caco-2 Cell Monolayers

The solid-state characteristics of pure IVMT-Rx-3 were investigated using powder X-ray diffraction (PXRD) and differential scanning calorimetry (DSC). PXRD measurements were conducted on a D8 Advance diffractometer (Bruker AXS Inc., Madison, WI, USA) operated at 40 mA and 40 kV using copper (Cu-Kα1) radiation (λ = 1.5418 Å). The drug powder was deposited on an adhesive sample holder with a thickness of 0.5 mm and analyzed over a 2θ range of 0° to 40° in step-scan mode at a scanning rate of 0.02°/second. Moreover, thermal behavior was assessed using a DSC Q1000 V9.9 Build 303 (TA Instrument Inc., New Castle, DE, USA). Approximately 1.5-2 mg of IVMT-Rx-3 was non-hermetically sealed in an aluminum pan and scanned at a heating rate of 5 °C/min over the temperature range of 25-300 °C under a nitrogen atmosphere.

The in vitro permeability of IVMT-Rx-3 across a Caco-2 cell monolayer was determined. Caco-2 cells at a density of 3 × 10^5^ cells/well were seeded onto 12-well Transwell^®^ filter inserts in complete Dulbecco’s modified Eagle medium (DMEM) and allowed to grow and differentiate for 21–29 days to form a confluent monolayer. The apical and basolateral compartments were then stabilized with 0.5 mL and 1.5 mL Hanks’ balanced salt solution (HBSS), respectively, at 37 °C for 20 min. To determine the apical (AP) to basolateral (BL) permeability of IVMT-Rx-3, HBSS in the AP compartment was replaced with 0.5 mL of IVMT-Rx-3 HBSS (200 µg/mL), and for the BL to AP permeability, HBSS in the BL compartment was replaced with 1.5 mL of IVMT-Rx-3 HBSS (200 µg/mL). The plate assembly was incubated at 37 °C. Then, 200 µL samples were either withdrawn from basolateral compartment or apical compartment and replaced with 200 µL fresh HBSS at 0.5, 1, 2, 3, 4, and 5 h. For both AP to BL and BL to AP permeability, three standard reference chemicals including metoprolol (a high-permeability BCS class I compound), erythromycin and cimetidine (both low-permeability BCS class III compounds) at a concentration of 10 µM were tested in parallel. The amount of IVMT-Rx-3 and each of the reference chemicals that permeated through the Caco-2 monolayer was quantified using HPLC-UV system (Shimadzu Prominence LC system; Shimadzu Corporation, Kyoto, Japan). The apparent permeability coefficients (P_app_) of IVMT-Rx-3, metoprolol, erythromycin, and cimetidine were calculated using the following equation:

P_app_ = dQ/dt × 1/(A × C_0_)

where dQ/dt is the steady-state flux (µg/s), C_0_ is the initial concentration of IVMT-Rx-3, metoprolol, erythromycin, or cimetidine on the apical side (µg/mL), and A is the surface area of the filter (cm^2^).

### 2.3. Preparation and Characterization of IVMT-Rx-3-MEs

#### 2.3.1. Selection of Formulation Components for IVMT-Rx-3-MEs

The selection of ME components is fundamentally driven by their ability to solubilize poorly water-soluble drugs, since adequate drug solubility within the formulation is critical to maximize drug loading, ensure long-term physicochemical stability, and enhance oral bioavailability. To determine the saturation solubility of IVMT-Rx-3 in different oils, surfactants or co-surfactants, and aqueous phases, an excess amount of IVMT-Rx-3 was added to 1 mL of oils (oleic acid, Capryol 90, and Maisine oil), surfactants or co-surfactants (Labrafil M 1944 CS, PEG 400, Tween 80, Transcutol HP, Labrasol, propylene glycol, and Kolliphor EL), and aqueous phases (deionized water and PBS, pH 7.4) in a glass vial. Each sample was vortexed and incubated in an isothermal shaker (100 rpm) maintained at 25 ± 1.0 °C for 48 h to attain equilibrium. Following incubation, the samples were centrifuged at 4000× *g* and 25 ± 1.0 °C for 15 min using an Eppendorf 5424 R Refrigerated Centrifuge (Eppendorf SE, Hamburg, Germany). The resulting supernatant was collected, diluted with acetonitrile, and filtered through a 0.22 μm polytetrafluoroethylene (PTFE, hydrophobic) membrane filter (Thermo Fisher Scientific, Waltham, MA, USA) prior to analysis. The concentration of IVMT-Rx-3 in the filtrates was quantified using HPLC-UV as described in Section 2.3.5. Following the selection of the excipients based on higher solubility of IVMT-Rx-3, miscibility studies were conducted as previously described. In brief, equal volumes (1 mL each) of the selected excipients were mixed, vortexed for 10 min, and allowed to equilibrate at room temperature for approximately 30 min. The mixtures were then visually examined for transparency, turbidity, and any evidence of phase separation.

#### 2.3.2. Formulation and Characterization of the O/W ME System

Based on solubility and miscibility studies, Capryol 90 was selected as the oil phase. A fixed weight ratio of Labrasol to Tween 80 (1:1 *w*/*w*) was used as the surfactant mixture. Five different co-surfactant systems were prepared by combining Transcutol HP and Vitamin E TPGS at varying weight ratios of 1:1, 2:1, 3:1, 4:1, and 5:1 (*w*/*w*). Phosphate-buffered saline (PBS, pH 7.4) was used as the aqueous phase. The drug was initially dissolved in 10% of the total PBS and mixed with the oil, surfactant, and co-surfactant mixture using vortex mixing. Upon formation of a monophasic system, the remaining PBS is gradually added under continuous vortex mixing for 10 min. The prepared emulsions were further sonicated using an ultrasonic Liquid Processor VCX 500 (Sonics & Materials, Inc., Newtown, CT, USA) at 40% amplitude for 3 min. Each IVMT-Rx-3-ME’s formulation was subsequently subjected to physicochemical characterization.

The average droplet size, polydispersity index (PDI), and zeta potential of the IVMT-Rx-3-ME were determined using Malvern Zetasizer Nano ZS90, Malvern Instruments, Malvern, UK. IVMT-Rx-3-ME formulations were diluted at a 1:100 (*v*/*v*) ratio using a 10 mM NaCl solution and sonicated for 1 min in a sonication bath (Branson CPX2800H; Branson Ultrasonics Corporation, Danbury, CT, USA) before measurement. The drug content in the IVMT-Rx-3-ME was quantified by diluting each sample with acetonitrile, followed by filtration through a 0.2 µm (PTFE, hydrophobic) membrane filter. The concentration of IVMT-Rx-3 was then quantified using a HPLC system with a UV detector, as described later in Section 2.3.5. The measured IVMT-Rx-3 concentration in the diluted sample (C_meas_) was corrected for dilution and compared with the theoretical concentration (C_theoretical_) based on the amount of drug added during formulation. Drug content was calculated as:

$$Drug\;content\left(\%\right)=\frac{C_{meas}\times DF}{C_{theoretical}}\times 100$$

where DF is the dilution factor.

In addition, the shape, droplet morphology, and droplet size of the selected IVMT-Rx-3-ME were determined by using an FEI Talos F200X G2 cryo-transmission (cryo-TEM) electron microscope (FEI Company, Hillsboro, OR, USA). IVMT-Rx-3-ME was diluted 100-fold with deionized water, and sample grids were prepared using an FEI Vitrobot Mark IV plunge freezer (FEI Company, Hillsboro, OR, USA). A 3.5 µL aliquot of the diluted formulation was applied to the grid, followed by blotting (blot force 5, 10 s blot time) and plunging into liquid ethane. Imaging was performed on a Thermo Scientific Glacios cryo-TEM (Thermo Fisher Scientific, Waltham, MA, USA) operated at 200 kV, equipped with an XFEG electron source and a Falcon 4 direct electron detector.

#### 2.3.3. In Vitro Permeation Studies Through a Parallel Artificial Intestinal Membrane

The in vitro artificial membrane permeabilities of IVMT-Rx-3 dispersed in PBS (pH 6.8), IVMT-Rx-3 dispersed in a mixture of 10% PEG 400 + 5% DMSO + 85% of 10% HP-β CD, and IVMT-Rx-3-MEs were determined by parallel artificial membrane permeability assay (PAMPA; BD BioSciences, San Jose, CA, USA), as described by supplier. Briefly, donor samples were prepared by diluting free IVMT-Rx-3, IVMT-Rx-3 dispersion, and IVMT-Rx-3-ME with PBS pH 6.8 to a final concentration of 200 µg/mL (based on IVMT-Rx-3). An aliquot of 200 µL of diluted samples was added to each well of the donor plate, while 300 µL of PBS pH 6.8 was added to each well of the acceptor plate. The donor plate was sandwiched with the acceptor plate, ensuring that the membrane of donor wells remained in direct contact with the media in the acceptor wells. The assembled system was then incubated at room temperature for 5 h. Following incubation, the donor and acceptor plates were detached, and samples were withdrawn from both the donor and acceptor plates. The concentration of IVMT-Rx-3 that permeated through the artificial membrane was measured using HPLC as described later. The effective permeability (P_e_) of each sample was calculated using the following formula: P_e_ = −ln(1 − C_A_[t]/C_equilibrium_)/(A × [1/V_D_ + 1/V_A_] × t) where P_e_ is the permeability (cm/s), A is the effective filter area (0.228 cm^2^), V_D_ is the volume of the donor well (0.2 mL), V_A_ is the volume of the receptor well (0.3 mL), t is the total time of incubation in seconds, C_A_(t) denotes the concentration of drug in the receptor well at time t, and C_equilibrium_ represents (C_D_[t] × V_D_ + C_A_[t] × V_A_)/(V_D_ + V_A_), where C_D_(t) denotes the concentration of drug in the donor well at time t.

#### 2.3.4. In Vitro Dissolution Study

To assess the dissolution profiles of free IVMT-Rx-3 dispersion (in 0.1 N HCl, pH 1.2 or PBS, pH 6.8) and selected IVMT-Rx-3-ME, in vitro drug release studies were performed in 100 mL of medium containing 0.1 N HCl (pH 1.2) or PBS pH 6.8 at 37 ± 0.2 °C using a USP I Apparatus (Basket) (Sotax AT Xtend; SOTAX Corporation, Westborough, MA, USA), rotating at 100 rpm. Hydroxypropyl methylcellulose (HPMC) capsules size #00 (Qualicaps^®^; Qualicaps, Inc., Whitsett, NC, USA) were filled with either IVMT-Rx-3 dispersion or IVMT-Rx-3-ME (equivalent to 6 mg IVMT-Rx-3 in 1.5 mL formulation). Each capsule was placed inside the basket and subjected to dissolution test, and 1 mL samples were withdrawn at 10, 20, 30, 45, 60, 90, 120, 180, 240, and 300 min. Meanwhile, the same volume of fresh medium was added to maintain a constant total volume. After filtration through 0.45 μm PVDF membranes, the amount of IVMT-Rx-3 in the dissolution medium was quantified by HPLC as described in Section 2.3.5.

#### 2.3.5. High-Performance Liquid Chromatography (HPLC) Quantification Method

Chromatographic separation of IVMT-Rx-3 was performed on an Agilent Pursuit XRs 5 C18 column (4.6 × 250 mm) using a mobile phase composed of acetonitrile and water containing 0.1% trifluoroacetic acid (TFA) in a 50:50 (*v*/*v*) ratio. The flow rate was maintained at 1.0 mL/min, with the column temperature set at 25 °C. The injection volume was 10 µL, and UV detection was performed at a wavelength of 284 nm.

#### 2.3.6. IVMT-Rx-3-ME Stability

The IVMT-Rx-3-ME formulation was selected based on the physicochemical characteristics and higher permeability and was subjected to a series of thermodynamic stability tests, including centrifugation, heating–cooling cycles, and freeze–thaw cycles. The impact of centrifugal force on potential phase separation of oil and water phases was evaluated by centrifuging IVMT-Rx-3-ME at 1000× *g* for 30 min using an Eppendorf 5424 R Refrigerated Centrifuge (Eppendorf SE, Hamburg, Germany). To understand the stability after heat–cool cycles, IVMT-Rx-3-ME was subjected to 6 cycles of heating (45 °C) and cooling (4 °C), followed by storage at room temperature for 48 h, after which it was visually examined. Similarly, freeze–thaw stability was assessed by subjecting IVMT-Rx-3-ME to three cycles of freezing at −20 °C and thawing at 25 °C, followed by 48 h storage at room temperature. After each test, the IVMT-Rx-3-ME was evaluated for physical instabilities, such as phase separation, creaming, and cracking.

To further evaluate the storage stability, IVMT-Rx-3-ME was stored in glass scintillation vials at 25 ± 5 °C for 12 months. At predetermined intervals, the formulation was analyzed for droplet size, PDI, zeta potential, and drug content. In addition, IVMT-Rx-3-ME was visually inspected for any signs of physical instability, including phase separation, turbidity, loss of transparency, precipitation, and color change. All stability studies were conducted in triplicates, and the results were reported as mean ± SD.

### 2.4. In Vivo Pharmacokinetic Study in Rats

Sprague Dawley rats (males, 250–300 g) were purchased from Charles River Laboratories (Wilmington, MA, USA) for performing the in vivo pharmacokinetic study with the approved protocol (AD10001739). The experimental animals were cared for by the VCU’s Department of Animal Resources. All animal experiments were performed in accordance with the NIH Guidelines regarding the Care and Use of Laboratory Animals and the guidelines of the IACUC.

The pharmacokinetic profiles of IVMT-Rx-3 dispersion prepared in 10% PEG 400 + 5% DMSO + 85% of 10% HP-β CD in water (vehicle) and IVMT-Rx-3-ME were evaluated following oral administration to rats to evaluate the impact of ME formulations on the oral absorption of IVMT-Rx-3. The above vehicle was selected to ensure uniform dispersion, facilitate dosing, and maintain excipient levels within ranges generally considered acceptable for preclinical use. The rats received either IVMT-Rx-3 dispersion or IVMT-Rx-3-ME at a dose of 20 mg/kg. Next, blood samples of 150 µL were collected via tail vein at pre-determined intervals, transferred into sodium EDTA-coated tubes, and immediately centrifuged (2500× *g*, 15 min, 4 °C). Plasma samples were separated and stored frozen at −80 °C until analysis.

To quantify the plasma concentration of IVMT-Rx-3, the plasma samples were thawed and centrifuged at 2500× *g* for 5 min at 4 °C. Subsequently, 100 µL of each standard solution or plasma sample was then mixed with 100 µL of internal standard (IS) solution (dexamethasone, 2.5 µg/mL). Then, 500 µL of ice-cold acetonitrile was added to precipitate protein. The mixture was vortex mixed and centrifuged at 9000× *g* for 5 min at 4 °C. Following centrifugation, the supernatant was transferred to a glass vial and dried using a sample concentrator dry-block (Techne, Cambridge, UK) maintained at 50 °C. The resulting residue was then reconstituted in 100 µL of acetonitrile:water (1:9, *v*/*v*). The IVMT-Rx-3 concentration was determined using Liquid Chromatography Mass Spectrometer (LC-MS-2020; Shimadzu Corporation, Kyoto, Japan) with a XBridge^®^ BEH C18 XP column (30 × 3.0 mm, 2.5 μm; Waters Corporation, Milford, MA, USA), and the samples were chromatographed under isocratic mobile phase consisting of acetonitrile with 0.1% formic acid and water with 0.1% formic acid (30:70, *v*/*v*) at a flow rate of 0.8 mL/min. A 10 μL aliquot of the sample was injected, and IVMT-Rx-3 was measured in positive ion mode using electrospray ionization (ESI). The optimized mass spectrometric parameters included an interface temperature of 350 °C, desolvation line (DL) temperature of 250 °C, nebulizing gas flow rate of 1 L/min, and drying gas flow rate of 10 L/min.

### 2.5. In Vivo Tumor Growth Inhibition Efficacy in Mice

All experimental metastatic and allograft experiments were conducted in accordance with the approved protocol by VCU IACUC (AM10183). For this purpose, two-month-old C57BL/6 mice were procured from Envigo Laboratories (Indianapolis, IN, USA). In the experimental metastasis model, 1 × 10^5^ B16 cells were administered intravenously (n = 10) and allocated into two groups: a control group receiving just oral vehicle ME and an IVMT-Rx-3-ME group treated three times weekly for two weeks. Animals were sacrificed after 15 days, lungs were removed, and total nodules were computed to assess anti-metastatic activity. In the allograft study, 5 × 10^5^ B16 cells were implanted subcutaneously in the left flank. Upon reaching approximately 100 mm^3^, the animals were randomly divided into four experimental groups: Group I: Control (Vehicle ME); Group II: IVMT-Rx-3-ME; Group III: Anti-PD-L1; and Group IV: IVMT-Rx-3-ME combined with anti-PD-L1 (Combination). Tumor dimensions were assessed using calipers every 2 to 3 days, and tumor volume was computed using the formula V = (length × width^2^)/2. Tumor growth curves were constructed for each group, and the data were presented as mean ± SEM.

### 2.6. Histopathology Evaluation

To assess the potential toxicity of IVMT-Rx-3-ME, C57BL/6 mice were orally administered IVMT-Rx-3-ME three times per week for two weeks and compared with a healthy control group. On day 15, animals were euthanized using CO_2_ inhalation followed by cervical dislocation. For histopathological analysis, tissues including the heart, liver, spleen, kidney, duodenum, jejunum, ileum, and lungs were collected, fixed in 10% neutral buffered formalin for 24 h, transferred to 70% ethanol, processed, paraffin-embedded, sectioned at 5 μm thickness, and stained with hematoxylin and eosin (H&E). For lung metastasis and treatment confirmation, representative H&E-stained lung sections were examined to confirm metastatic tumor involvement, and semi-quantitative histological assessment was performed by measuring the proportion of lung involved by melanoma (tumor area/total lung area, %), proportion of necrosis (necrotic tumor area/total tumor area, %), greatest dimension of the single largest nodule (mm), and proportion of pigmented tumor (%).

### 2.7. Statistical Analysis

Behavioral data are expressed as mean ± SEM (Standard Error of the Mean). Time-course data and other behavioral results were analyzed using one-way or two-way repeated measures of variance (ANOVA) followed by post hoc Tukey test with the alpha level set at 0.05. The behavioral statistical analysis was performed with GraphPad Prism software, version 9.5. The probability was considered significant if *p* < 0.05. No significant sex differences were observed in any of the experiments, so the data from both males and females were pooled. For all other analysis, one-way or two-way ANOVA followed by Tukey’s multiple-comparison test was used to compare more than two mean values. All data were expressed as mean ± SD for in vitro analysis and mean ± SEM for in vivo analysis. In all analyses, *p* < 0.05 was considered statistically significant.

## 3. Results

### 3.1. Synthesis and Primary Characterization of IVMT-Rx-3

The synthesis scheme of IVMT-Rx-3 and primary characterization data, a docking model, target binding and PK data have been reported previously [20]. In this study, we used IVMT-Rx-3 from the same synthesis batch used in [20].

Solid-state characterization of IVMT-Rx-3 was performed to establish the API’s baseline physical form, since crystalline/amorphous nature and thermal transitions can directly influence dissolution behavior, physical stability, and precipitation or recrystallization propensity during processing and in the dynamic GI pH environment.

**Figure 1:**
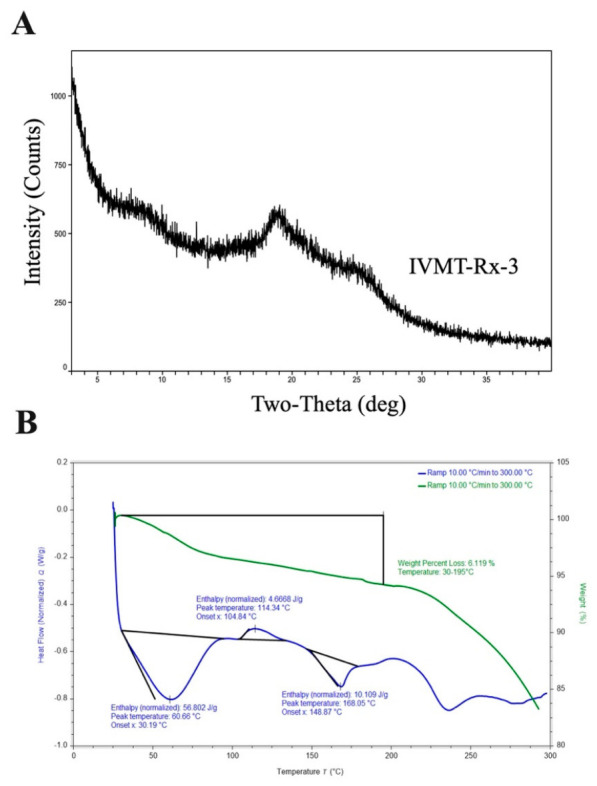
(**A**) PXRD pattern and (**B**) DSC thermogram of IVMT-Rx-3.

The PXRD analysis of IVMT-Rx-3 exhibited a broad diffuse halo without distinct Bragg reflections, indicating the absence of long-range crystalline order and confirming the amorphous nature of the drug (Figure 1A). The DSC thermogram showed multiple broad endothermic events without a sharp melting endotherm (Figure 1B), further consistent with an amorphous material. A broad endotherm centered at ~60 °C was observed, consistent with loss of adsorbed moisture or residual solvent as also reflected by the accompanying mass loss in the TGA trace. A weaker event near ~110–115 °C was detected, which may reflect amorphous structural relaxation. Importantly, a broader endothermic transition with an onset around ~148 °C (peak ~168 °C) was observed, which can be related to enthalpy relaxation, structural rearrangement or the onset of thermal degradation rather than a true crystalline melting event. Overall, the absence of a distinct melting peak supports that IVMT-Rx-3 is predominantly amorphous.

**Figure 2:**
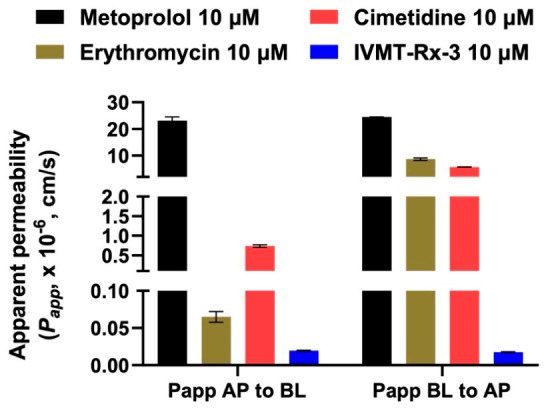
Average apparent permeability of IVMT-Rx-3, metoprolol, cimetidine, and erythromycin at 10 µM loading concentration across Caco-2 cells’ monolayer. Each value represents the mean ± standard deviation (n = 3).

The intestinal permeation profile of IVMT-Rx-3 was analyzed by evaluating apparent permeability coefficients (P_app_) through the Caco-2 cell monolayer experiment (Figure 2). The P_app_ values for IVMT-Rx-3 in the apical-to-basolateral (AP→BL) and basolateral-to-apical (BL→AP) directions were determined and found to be 0.019 × 10^6^ cm/s and 0.018 × 10^6^ cm/s, respectively. For comparative analysis, three standard reference chemicals (i) metoprolol (a high-permeability BCS class I compound), (ii) erythromycin, and (iii) cimetidine (both low-permeability BCS class III compounds) were included in this experiment. Metoprolol is commonly used to establish the high-permeability threshold due to its complete absorption and passive transcellular transport characteristics. Erythromycin and cimetidine serve as benchmarks for low permeability and are recognized substrates for efflux transporters like the P-glycoprotein. The reference compounds demonstrated markedly elevated P_app_ values in both transport directions relative to IVMT-Rx-3, indicating that IVMT-Rx-3 possesses restricted permeability and may be influenced by active efflux mechanisms. Given the amorphous nature of IVMT-Rx-3 and the absence of a defined melting point, potential challenges related to physical stability and dissolution behavior were anticipated. Furthermore, the low apparent permeability observed in the Caco-2 model suggests limited intestinal absorption. Accordingly, a ME system was proposed as a formulation strategy to further enhance drug content, stabilize amorphous drug, and improve intestinal permeability.

### 3.2. Formulation Preparation and Characterization

#### 3.2.1. Solubility of IVMT-Rx-3 in Various ME Components

**Figure 3:**
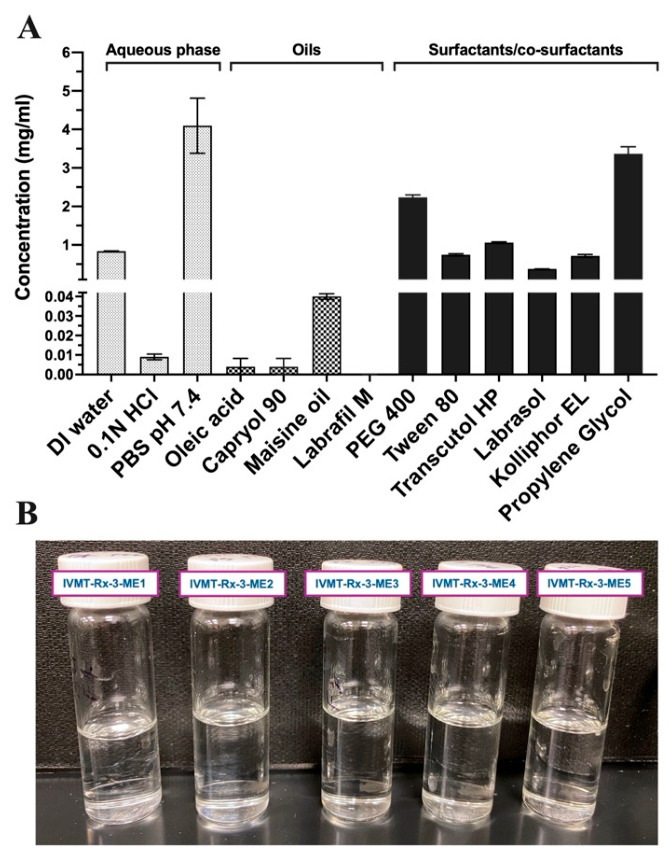
(**A**) Solubility of IVMT-Rx-3 in various oils, surfactants, and co-surfactants. (**B**) Representative pictures of IVMT-Rx-3-MEs. Each value represents the mean ± standard deviation (n = 3).

To find appropriate oils, surfactants, and co-surfactants for formulating a stable ME, the solubility properties of IVMT-Rx-3 were assessed. IVMT-Rx-3 has low solubility in oils, with solubility values of 0.04 mg/mL in Maisine oil, 0.004 mg/mL in oleic acid, and 0.004 mg/mL in Capryol 90 (Figure 3A). Despite its limited solubility, Capryol 90 was chosen for its comprehensive miscibility with other formulation components. The solubility of IVMT-Rx-3 in various surfactants differed, being highest in propylene glycol (3.36 mg/mL) compared to Transcutol HP (1.06 mg/mL), Labrasol (0.37 mg/mL), and Tween 80 (0.74 mg/mL). These surfactants have been tested for their compatibility with Capryol 90. IVMT-Rx-3 exhibited superior solubility in PBS at pH 7.4 (4.10 mg/mL) and has been selected as the aqueous component. Based on these solubility findings, the final IVMT-Rx-3 concentration in the optimized ME was set at 4 mg/mL.

#### 3.2.2. IVMT-Rx-3-ME Formulation and Development

**Figure 4:**
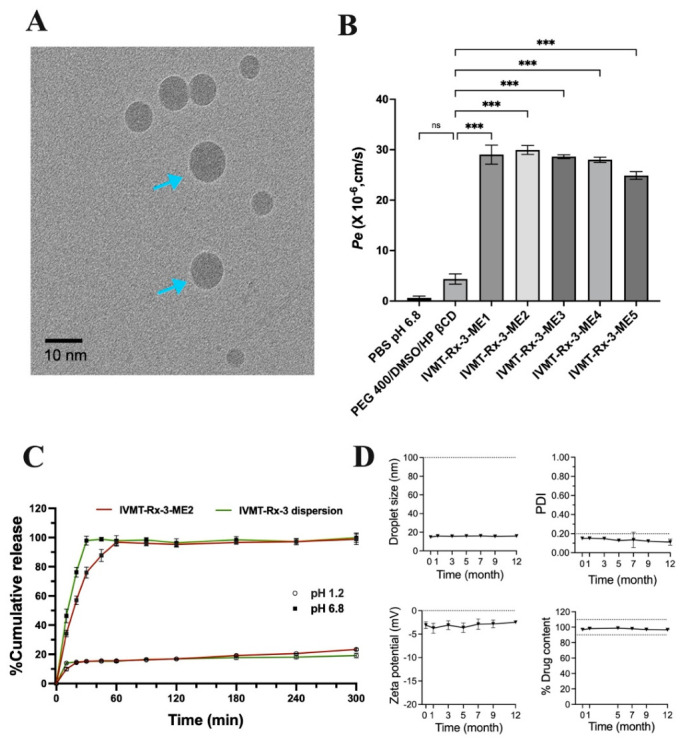
(**A**) Representative Cryo-TEM image of IVMT-Rx-3-ME2. Blue arrows show drug loaded droplets in the ME system (**B**) Effective permeability of IVMT-Rx-3 dispersion (in PBS, pH 6.8 and in a mixture of 10% PEG 400 + 5% DMSO + 85% of 10% HP-β CD) or IVMT-Rx-3-ME1-5 (one-way ANOVA followed by Tukey’s multiple comparison test) (mean ± SD; n = 6). (**C**) In vitro cumulative percentage release profiles of IVMT-Rx-3 dispersion or IVMT-Rx-3-ME2 in pH 1.2 and pH 6.8 media (mean ± SD; n = 3). (**D**) Storage stability of IVMT-Rx-3-ME at 25 ± 5 °C. Average droplet size, Polydispersity index (PDI), Zeta potential, and Drug content of IVMT-Rx-3-ME2 (mean ± SD; n = 3). Dotted reference lines in (**D**) indicate acceptance thresholds for ME droplet size, acceptable PDI, neutral zeta potential (~0 mV), and acceptable drug-content variation (±15%). *** *p* < 0.001; ns, non-significant.

Based on solubility data, Capryol 90, Labrasol and Tween 80, and Transcutol HP were chosen as the oil, surfactant, and co-surfactant, respectively. Furthermore, Vitamin E TPGS was used as a secondary co-surfactant. To utilize its excellent aqueous solubility in PBS (pH 7.4), IVMT-Rx-3 was initially diluted in a minimal volume of PBS (pH 7.4) and subsequently, Capryol 90, Labrasol, Tween 80, and Transcutol HP were included, together with an additional amount of PBS at pH 7.4, to create each of the liquid MEs (Figure 3B). To enhance the formulation and create a thermodynamically stable composition, Transcutol HP and Vitamin E TPGS were evaluated at varied ratios across five distinct formulations (IVMT-Rx-3-ME1-5), as illustrated in Table 1. Figure 4A presents the cryo-TEM image of the IVMT-Rx-3-ME2, validating the existence of spherical and uniformly dispersed nanoscale droplets. The observed morphology and size were congruent with measurements obtained using dynamic light scattering, corroborating the establishment of a stable ME system.

#### 3.2.3. Physicochemical Characterization

As shown in Table 1, IVMT-Rx-3-ME1 exhibited the smallest droplet size of the five formulations, showing an average diameter of 9.1 ± 0.1 nm. However, the other formulations showed very comparable droplet sizes, ranging from 9.4 ± 0.1 nm (IVMT-Rx-3-ME2) to 10.4 ± 0.3 nm (IVMT-Rx-3-ME5). The formulations also exhibited good polydispersity and thermodynamic stability, with PDI and zeta potential values ranging from 0.03 ± 0.02 to 0.08 ± 0.007 and −1.12 ± 0.2 mV to −3.89 ± 1.1 mV, respectively. Additionally, each of the 5 ME formulations showed a drug content of >90%. Based on the physicochemical results, each formulation was further characterized for permeability.

#### 3.2.4. In Vitro Permeability Evaluations

To evaluate the passive diffusion characteristics of IVMT-Rx-3, we performed a PAMPA study using a PVDF membrane coated with phospholipids, which mimics the lipid composition of the intestinal epithelium. The study compared the permeation of the free drug, IVMT-Rx-3 dispersion, and five ME formulations (Table 1). The dispersion exhibited approximately a 4-fold increase in effective permeability (P_e_) compared to the free drug, indicating improved solubilization and diffusivity. However, all ME formulations exhibited significantly higher P_e_ values than the dispersion, with at least a 5.7-fold increase in permeation (Figure 4B). Among the five tested MEs, IVMT-Rx-3-ME2 exhibited the highest permeability (P_e_ = 29.96 × 10^6^ cm/s). In combination with the physicochemical results, this result identified IVMT-Rx-3-ME2 as the leading formulation, and it was therefore selected for further in vivo pharmacokinetic and efficacy evaluations.

#### 3.2.5. Dissolution Study

To evaluate the drug release behavior of IVMT-Rx-3 under physiological conditions, an in vitro dissolution study was conducted using free IVMT-Rx-3 dispersion and the optimized ME (IVMT-Rx-3-ME2) in 0.1 N HCl (pH 1.2) and phosphate buffer (pH 6.8) (Figure 4C). In 0.1 N HCl, approximately 18% of the drug was released from the ME within 60 min, which was comparable to the release from the IVMT-Rx-3 dispersion. This limited release at acidic pH is consistent with the intrinsic solubility profile of IVMT-Rx-3, as previously described.

In contrast, at pH 6.8, drug release was substantially enhanced for both the IVMT-Rx-3-ME2 and IVMT-Rx-3 dispersion, with nearly 97% of the total drug released within 60 min (Figure 4C). This finding also aligns with the solubility results, indicating that IVMT-Rx-3 is highly soluble in near-neutral pH condition. The similarity in dissolution profiles between the ME and dispersion at pH 6.8 suggests that the ME formulation does not compromise the intrinsic solubility of IVMT-Rx-3. Instead, the primary advantage of the ME is proposed to be its ability to enhance intestinal permeability, as demonstrated in the PAMPA and Caco-2 assays, rather than to improve dissolution rate.

### 3.3. Storage Stability of IVMT-RX-3-ME2

The thermodynamic stability of IVMT-Rx-3-ME2 is attributed to the optimized combination of surfactants and co-surfactants, which markedly reduces interfacial tension and promotes the formation of robust interfacial film between the oil and aqueous phases. IVMT-RX-3-ME2 successfully passed the thermodynamic stress testing, including freeze-thaw cycling, centrifugation, and heat-cool cycles, without evidence of turbidity, phase separation, precipitation, or decrease in drug content. Long-term storage stability of the formulation at 25 ± 2 °C for 12 months showed no visible signs of physical instability, such as creaming, turbidity, phase separation, or precipitation, indicating excellent physical stability. Furthermore, the mean hydrodynamic droplet size, PDI, zeta potential, and drug content remained statistically unchanged compared to the freshly prepared formulation (Figure 4D), demonstrating strong resistance to droplet coalescence and Ostwald ripening, which are recognized as the dominant mechanisms of microemulsion destabilization and phase separation.

### 3.4. In Vivo Pharmacokinetics of IVMT-Rx-3-ME2

**Figure 5:**
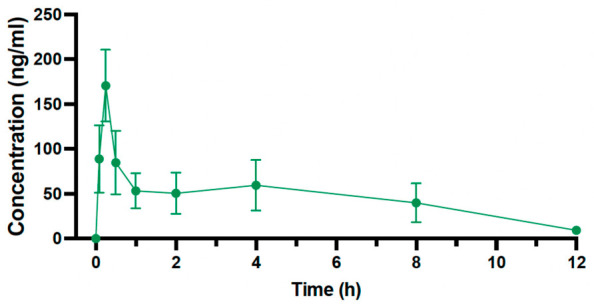
Plasma concentration–time profiles of IVMT-Rx-3-ME2 after oral administration of 20 mg/kg in rats (mean ± SEM, n = 4). Non-formulated IVMT-Rx-3 dispersion was not detected.

The in vivo absorption study was performed to determine whether the IVMT-Rx-3-ME2 formulation enhances absorption and oral bioavailability compared to IVMT-Rx-3 dispersion. Following oral administration in rats, the plasma concentration–time profiles of IVMT-Rx-3 were evaluated for both the ME and control (dispersion) groups (Figure 5). In the control group, plasma concentrations of IVMT-Rx-3 remained below the LOQ (1 ng/mL) throughout the study, indicating negligible systemic absorption of the free drug. In contrast, the IVMT-Rx-3-ME2 exhibited a sustained plasma concentration profile, confirming that ME-based delivery significantly improves the oral absorption of IVMT-Rx-3. Pharmacokinetic parameters following oral administration of IVMT-Rx-3-ME2 and control dispersion (20 mg/kg) are summarized in Table 2. The IVMT-Rx-3-ME2 achieved a good systemic exposure, with AUC_last_ and AUC_inf_ values of 526.4 ± 461.0 ng·h/mL and 573.5 ± 492.8 ng·h/mL, respectively. In contrast, plasma concentrations in the control group remained below the limit of quantification throughout the study, precluding calculation of any pharmacokinetic parameters. For the IVMT-Rx-3-ME2 group, the C_max_ was 170.5 ± 79.9 ng/mL, reached at a median T_max_ of 0.25 ± 0.00 h, indicating rapid absorption. The T_1/2_ was estimated at 3.2 ± 0.98 h. These data suggest that the ME formulation significantly enhances the oral bioavailability of IVMT-Rx-3 compared to the unformulated solution.

### 3.5. In Vivo Anti-Metastatic and Antitumor (In Combination with Anti-PD-L1) Effect of Orally Administered IVMT-RX-3-ME2

**Figure 6:**
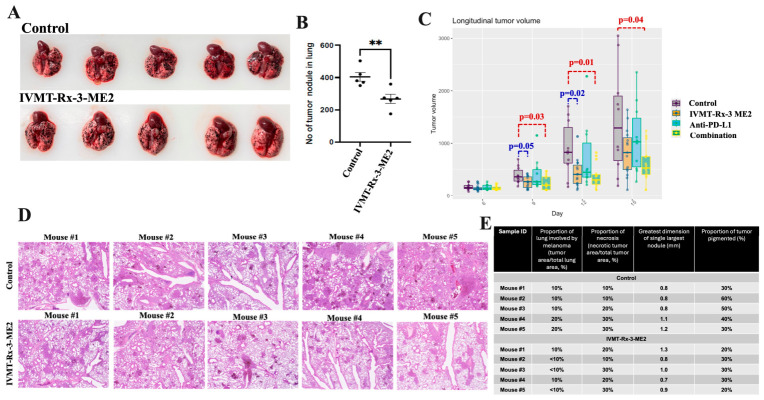
IVMT-Rx-3-ME demonstrates antitumor and anti-metastatic effects in an experimental murine model. B16 cells (1 × 10^5^ cells) were administered via the tail vein of C57BL/6 mice to generate lung metastases (n = 5, per group). On the second day, mice were divided into two groups: The Vehicle group (ME without IVMT-Rx-3) and the IVMT-Rx-3-ME2 group. Mice received oral administration of 30 mg/kg of IVMT-Rx-3-ME2 on alternate days for a total of six treatments over a two-week period. Lungs were removed, and nodules were recorded to assess the metastatic load. (**A**) Representative lung imaging on day 15; (**B**) quantification of metastatic nodules (mean ± SEM). (**C**) to assess the antitumor efficacy, B16 cells were subcutaneously inoculated into C57BL/6 mice, and upon the emergence of visible tumors, the animals were allocated into four experimental cohorts (n = 10, per group). Control, IVMT-Rx-3-ME2, Anti PD-L1, and IVMT-Rx-3-ME2 combined with Anti PD-L1. Graphical representation of the tumor volume at various time points (days). (**D**) Representative H&E-stained histological sections of lungs at day 15, photomicrographs of the 40× fields with greatest density of tumor observed per case. (**E**) Quantitative histopathological assessment of H&E-stained lung sections. Statistical analysis and the associated *p*-values were reported at various time points. *p* > 0.05 or non-significant results were not reported. ** *p* < 0.01.

The anti-metastatic efficacy of orally administered IVMT-Rx-3-ME2 was first evaluated using the B16 experimental lung metastasis model. Treatment with IVMT-Rx-3-ME2 significantly inhibited lung metastases compared to the control group (Figure 6A). Quantification of metastatic nodules confirmed a significant reduction in lung tumor nodules in the IVMT-Rx-3-ME2-treated group compared with control mice (*p* < 0.01; Figure 6B). The antitumor activity of IVMT-Rx-3-ME2 was further examined in the B16 subcutaneous allograft model, either alone or in combination with anti-PD-L1 antibody. Oral IVMT-Rx-3-ME2 monotherapy showed moderate inhibition of tumor growth over the treatment period, whereas anti-PD-L1 alone showed limited efficacy. Notably, the combination of IVMT-Rx-3-ME2 with anti-PD-L1 resulted in better tumor growth suppression compared with either treatment alone, supporting the potential benefit of combining oral IVMT-Rx-3-ME2 with anti-PD-L1 (after 15 days, the end point of this study, i.e., there is a ~50% suppression compared to the control group) (Figure 6C).

### 3.6. Histological Evaluation

Histopathological evaluation of H&E-stained lung sections further confirmed the presence of melanoma metastases in the lung tissue at day 15 (Figure 6D). Based on semi-quantitative histologic assessment mice, treated with IVMT-Rx-3-ME2, showed, on average and subjectively, a lower proportion of lung areas involved by melanoma compared with the control group (Figure 6E). Additional pathological assessment, including tumor necrosis, greatest dimension of the single largest nodule, and tumor pigmentation were not varied. It should be noted that these metastatic lesions may have developed distinct molecular signatures as they grew and persisted during treatment. To investigate that, more research is required.

**Figure 7:**
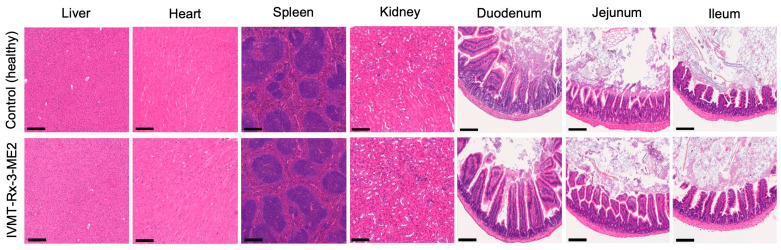
Representative H&E-stained histological sections of major organs (liver, heart, spleen, kidney, duodenum, jejunum, and ileum) collected after oral administration of vehicle ME (control) or IVMT-Rx-3-ME2 (30 mg/kg, on alternate days; six total doses over 2 weeks). Scale bar: 200 μm.

Examination of major organs, including the liver, heart, spleen, kidney, and small intestine (duodenum, jejunum, and ileum), revealed no treatment-related abnormalities after 14 days of oral IVMT-Rx-3-ME2 administration compared with healthy controls (Figure 7). Liver sections showed preserved hepatic architecture with intact lobular structure and no evidence of inflammatory cell infiltration, hepatocellular degeneration, or necrosis. Cardiac tissue displayed normal myocardial fiber alignment without signs of fibrosis, myocyte hypertrophy, or structural disruption. The spleen maintained normal structure with clearly demarcated red and white pulp regions. Kidney sections demonstrated intact glomeruli and tubular structures with no evidence of tubular injury, interstitial inflammation, or necrotic changes. Consistent with systemic tolerability, the duodenum, jejunum, and ileum exhibited preserved mucosal architecture without clinically significant epithelial damage, villus blunting, or inflammatory alterations at the administered dose.

## 4. Discussion

Despite excellent bioactivity, when delivered through the intraperitoneal route, the clinical translational potential of IVMT-Rx-3 is limited due to poor oral bioavailability. Oral chemotherapy offers several advantages over other routes of administration, including improved patient compliance, and increased adherence to long-term treatment regimens due to the ease of self-administration [27]. However, the effective oral delivery of anticancer agents is often hindered by factors such as poor solubility, limited intestinal permeability, gastrointestinal instability, and extensive pre-systemic metabolism [28,29]. These barriers can lead to subtherapeutic drug levels, necessitating higher or more frequent dosing, which in turn may increase the risk of systemic toxicity and adverse side effects [30]. In addition, solid-state characterization of IVMT-Rx-3 (Figure 1) indicated a predominantly amorphous profile, which is relevant to its oral delivery performance. Because the amorphous state is a higher-energy and less stable form, it may dissolve rapidly and transiently generate supersaturated concentrations that are thermodynamically unstable and prone to recrystallization or precipitation as GI conditions change. Consistent with this behavior, these findings further support the rationale for selecting a ME platform to maintain IVMT-Rx-3 in a solubilized state and mitigate precipitation-driven variability, thereby improving oral absorption.

In the current study, we were able to develop an oral ME which successfully solubilized and encapsulated IVMT-Rx-3 using biocompatible oils, surfactants, and co-surfactants (Table 1). Initial solubility screening of excipient candidates (Figure 3A) revealed that IVMT-Rx-3 was highly soluble in surfactant components but showed limited solubility in oil components. This is likely attributed to the amphiphilic nature of the surfactants [31]. This posed a significant challenge in formulation of the MEs, as successful ME formulation typically relies on the ability to first solubilize the compound in the oil phase [31,32]. However, IVMT-Rx-3 showed high aqueous solubility, particularly in PBS pH 7.4. When formulating, we were able to leverage this by first dissolving the drug in a small quantity of total PBS, then continuing with the addition of the other formulation components. After ensuring the IVMT-Rx-3 was completely dissolved in the system through sonication, the remaining amount of PBS was added. Further, selection of the oil, surfactants and co-surfactants was not solely dependent on the magnitude of IVMT-Rx-3 solubility in each component. While the results suggest that the use of Maisine oil, PEG 400, and propylene glycol may be promising (Figure 3A), compatibility of excipients with one another was the driving force behind the final composition choice. Because ME systems rely on the ability of the surfactants to reduce the surface tension between the oil and aqueous phases, good miscibility and stability between the oil and surfactants must be balanced with their ability to solubilize the drug compound [33]. Based on these criteria, Capryol 90, Labrasol, Transcutol HP, and Tween 80 were selected. Not only have these three excipients been used in similar formulations, but they offer some distinct therapeutic advantages [34,35,36]. Capryol 90 is often employed due to its high biocompatibility, Labrasol for its exceptional intestinal permeation enhancement, and Tween 80 for its minimized sensitivity to pH fluctuations and high ionic strength [37,38,39]. Although not discretely tested in the solubility analysis, we also decided to use Vitamin E TPGS (a water-soluble derivative of Vitamin E) as a secondary co-surfactant, hoping to draw upon its ability to further increase intestinal permeability while also acting as a P-glycoprotein (P-gp) inhibitor in the formulation [40,41].

After selecting the excipients and developing the formulation procedure, we were able to create five different MEs, each with varying ratios of the two co-surfactants, Transcutol HP and Vitamin E TPGS. The total volume of co-surfactants, however, remained constant at 40% *v*/*v* across the formulations, as did the oil and surfactant compositions (5% *v*/*v* and 12% *v*/*v*, respectively). The formulations were then characterized and compared to identify which of the five ME formulations had the desired properties—a droplet size < 100 nm, PDI < 0.2, near-neutral zeta potential (−10 mV to +10 mV), and excellent drug loading (>90%). A primary important parameter in formulation development is droplet size, as it can profoundly influence lipolysis, drug release, and absorption in the GI tract [42]. A smaller size is desired in order to increase the overall surface area which comes into contact with the intestinal membranes, supporting better absorption and ultimately increasing the oral bioavailability of the drug compound [42,43]. Physicochemical characterization revealed that all formulations had relatively similar droplet sizes. Additionally, the PDI is a normalized measure of how uniform the droplet sizes within the emulsion are, with a PDI closer to 0 being desired [44]. For each of the five formulations, PDI values were also similar and very small, indicating emulsion uniformity and a homogenous distribution of droplet sizes across the board. Zeta potential, or surface charge, is closely related to the stability of the formulation, and also impacts how the ME performs after oral administration. Due to the negative charge associated with the GI membrane, the desired ME formulation should have a near-neutral charge, supporting its ability to interact with and permeate through the membrane [45]. Physicochemical characterization also revealed very similar zeta potentials among the formulations. Finally, loading efficiency of IVMT-Rx-3 into each of the MEs was assessed. All five formulations showed acceptable drug loading (exceeding 90%), but there were some differences. Considering these four physicochemical properties, a leading formulation could not be identified. Ultimately, quantification of in vitro permeability was needed to distinguish the best ME composition.

ME droplets are nanometer-sized and inherently spherical in morphology, a feature that has been linked to enhanced cellular uptake and drug delivery efficiency. Cryo-TEM confirms that these lipid-based droplets are nearly spherical and uniformly dispersed, allowing intimate contact with cell membranes. Their small size (often 10–100 nm) and high surface-area-to-volume ratio facilitate endocytic uptake, yielding greater intracellular drug accumulation compared to free drug solutions [46]. For example, genipin encapsulated in ~16 nm ME droplets showed significantly higher uptake in Caco-2 cells and improved bioavailability relative to unformulated genipin [46]. Similarly, a disulfiram-loaded ME achieved markedly enhanced cellular internalization and cytotoxic efficacy in cancer cell lines versus the free drug [47].

Initial in vitro permeability studies using the Caco-2 cell monolayer model revealed that IVMT-Rx-3 exhibited low apparent permeability (Figure 2), likely due to limited transcellular transport and potential involvement of active efflux mechanisms such as P-gp-mediated transport, as observed with other poorly permeable chemotherapeutic agents [48,49]. To address these limitations and enhance oral absorption, an oil-in-water ME formulation was developed, aiming to improve membrane permeation and bypass efflux-related barriers.

MEs have been widely reported to enhance the intestinal absorption and oral bioavailability of hydrophilic and poorly permeable drugs by multiple mechanisms [23,50,51]. Consistent with our previous studies demonstrating that formulation components such as Labrasol and Transcutol HP enhance epithelial permeability, the non-ionic surfactants used in the IVMT-Rx-3 formulation may transiently disrupt intestinal tight junctions, thereby promoting paracellular transport [48]. In Caco-2 monolayers, Labrasol at low concentrations (0.1–1% *v*/*v*) increased the flux of a paracellular marker (mannitol) by ~4.6- to 33-fold via reorganization of F-actin and ZO-1 tight-junction proteins [50]. Likewise, Tween 80 at sub-cytotoxic levels (~0.2% *w*/*v*) reduced transepithelial resistance and doubled the flux of a fluorescent tracer, indicating loosening of the tight junction barrier [50]. In addition, Vitamin E TPGS, an amphiphilic surfactant used in the formulation, is known to inhibit P-gp, a major efflux transporter that limits oral drug absorption [52]. However, these mechanisms were not directly evaluated in the present study (e.g., TEER measurements or transporter inhibition assays).

MEs also maintain the drug in a solubilized state throughout the gastrointestinal tract, sustaining a high concentration gradient across the intestinal epithelium and thereby facilitating passive diffusion. Furthermore, upon digestion by pancreatic lipases, the oil components of MEs can form mixed micelles with free fatty acids and bile cholesterol, which help prevent drug precipitation and prolong the solubilized state in the intestinal lumen, supporting enhanced drug absorption [53]. In one study, a high-Labrasol ME was able to retain a hydrophilic antibiotic (gentamicin) in micellar form—at 25% Labrasol, gentamicin became non-dialyzable, indicating it remained associated with surfactant micelles rather than precipitating out [34]. In our study, PAMPA-based permeability evaluations further supported these mechanistic insights, demonstrating that formulation of IVMT-Rx-3 into a ME enhanced its effective permeability by at least 6-fold compared to both the free drug and the drug suspension (Figure 4B). This improvement underscores the ability of MES to facilitate passive transmembrane diffusion by maintaining the drug in a solubilized state and sustaining a favorable concentration gradient across the artificial membrane.

Hydrophilic drugs generally dissolve readily in aqueous media; therefore, formulating them into a ME is not intended to boost their dissolution rate, but rather to improve absorption and bioavailability. In vitro dissolution studies remain important to confirm that the ME does not impede drug release. In fact, ME-based formulations of hydrophilic compounds often show nearly complete and immediate drug release, comparable to the free drug in solution [50]. This unchanged dissolution profile is a desirable outcome because it means the formulation is not introducing a new rate-limiting step. For hydrophilic, poorly permeable drugs, the primary benefit of a ME lies in enhancing membrane transport and bioavailability, rather than in accelerating dissolution [54]. For example, fexofenadine (a highly water-soluble, low-permeability) achieved about a 3.8-fold higher oral bioavailability when delivered in a water-in-oil ME compared to a simple syrup solution, while dissolution was not affected in either case [54]. Similarly, an alendronate-loaded ME showed no disadvantage in dissolution but led to significantly enhanced transdermal permeation and roughly doubled bioavailability in vivo compared to pure drug solution [55]. Our dissolution study confirmed that IVMT-Rx-3-ME2 did not compromise the drug’s release behavior. At pH 6.8, both the IVMT-Rx-3-ME2 and the dispersion exhibited rapid and nearly complete release within 60 min, indicating that the formulation preserved the intrinsic solubility and fast dissolution characteristics of IVMT-Rx-3. These findings indicate that the ME maintained the drug in a solubilized state without introducing a release barrier.

The o/w ME markedly improved IVMT-Rx-3 oral uptake, yielding a higher C_max_ and AUC (compared to the drug dispersion in a mixture of 10% PEG 400 + 5% DMSO + 85% of 10% HP-β CD) with a rapid T_max_ (~0.25 h). Plasma concentrations of IVMT-Rx-3 in the control (free drug dispersion) group remained below the LOQ (1 ng/mL), indicating negligible systemic absorption. Such outcomes reflect multiple cooperative mechanisms, as discussed earlier. Notably, a slight elevation of plasma drug concentration observed at ~4 h may indicate enterohepatic recirculation. It is known that drugs absorbed and then excreted into bile can be re-released into the gut upon gallbladder contraction (often postprandially), resulting in secondary plasma peaks. The surfactant-induced formation of mixed micelles with bile salts may facilitate the resolubilization and reabsorption of IVMT-Rx-3, thereby prolonging systemic exposure. These findings align with in vivo studies of ME formulations and underscore the importance of further mechanistic investigations. For example, a bile-duct ligation study could confirm the role of bile-mediated recycling in the ME’s absorption profile, and comparisons between fed and fasted dosing could elucidate the influence of dietary stimuli (bile flow, gut motility) on drug uptake performance. Although the nominal mg/kg doses differ between rat PK (20 mg/kg) and mouse efficacy (30 mg/kg), BSA-normalized scaling (FDA Km method) indicates these regimens are broadly comparable (rat: ~120 mg/m^2^; mouse: ~90 mg/m^2^). A matched PK assessment in the efficacy model will be required to enable a formal quantitative PK/PD correlation.

Finally, IVMT-Rx-3-ME2 is effective in aggressive melanoma metastasis and demonstrated antitumor activity, particularly when combined with a checkpoint inhibitor. This approach leads to further refinement of the formulation with dosing and frequency optimization to obtain the maximum outcome. Our prior study rationalized the application of IVMT-Rx-3 and anti-PD-L1, which majorly impact the tumor microenvironment and enhance the immune cell infiltration and T cell cytotoxicity. Additionally, as a monotherapy, IVMT-Rx-3 could reduce the metastasis and thus collectively enhance the probability of survival. Although not tested yet, this approach could be equally impactful in diversified solid malignancies and associated metastases, particularly where MDA-9/syntenin is relevant in disease progression.

These efficacy outcomes are further supported when viewed alongside the systemic exposure achieved by oral IVMT-Rx-3-ME2. Oral administration of IVMT-Rx-3-ME2 achieved rapid absorption and substantial systemic exposure, whereas plasma IVMT-Rx-3 levels in the dispersion control group remained below the limit of quantification. This enhanced exposure corresponded with robust antitumor and anti-metastatic effects observed in the melanoma models. Furthermore, combining IVMT-Rx-3-ME2 with anti-PD-L1 checkpoint blockade yielded greater tumor suppression than either therapy alone. While not establishing a causal relationship, these findings suggest a positive correlation between the elevated systemic exposure provided by the ME and its therapeutic efficacy in vivo. However, matched PK sampling within the efficacy model is required to enable a quantitative PK/PD correlation. Repeated oral administration of IVMT-Rx-3-ME2 did not induce detectable pathological changes in the liver, heart, spleen, kidney, or intestinal tissues, indicating good systemic and gastrointestinal tolerability at the tested dose. While these results indicate a favorable safety profile, future studies involving extended dosing regimens will be required to fully assess chronic exposure and potential off-target effects.

## 5. Conclusions

In this study, IVMT-Rx-3-ME was developed for oral metronomic delivery, which effectively overcame intrinsic limitations of the drug such as negligible intestinal permeability and oral bioavailability. The optimized IVMT-Rx-3-ME exhibited high content, favorable physicochemical stability, and significantly improved permeability and dissolution compared to the free drug. These improvements translated into sustained systemic exposure in vivo which resulted in robust antitumor and anti-metastatic efficacy in murine melanoma models following oral administration. Notably, oral IVMT-Rx-3-ME synergized with immune checkpoint blockade (anti-PD-L1) to further suppress tumor growth without added toxicity. Collectively, these findings establish IVMT-Rx-3-ME as a potent and translationally feasible oral metronomic chemotherapy platform for metastatic melanoma and support further clinical development of MDA-9/Syntenin-1 targeted therapy, both as a monotherapy and in combination with immunotherapy.

## Figures and Tables

**Table 1:** Composition of IVMT-Rx-3-ME formulations and their mean droplet size, PDI, zeta potential, and drug content. Each value represents the mean ± standard deviation (n = 3).

Formulation Details		IVMT-Rx-3-ME1	IVMT-Rx-3-ME2	IVMT-Rx-3-ME3	IVMT-Rx-3-ME4	IVMT-Rx-3-ME5	Volume
		Ratio					
**Oil**	Capryl 90	1					5%
**Emulsifier**	Labrasol/Tween 80	1:1					12%
**Co-emulsifier**	Transcutol P/Vit E TGPS	1:1	2:1	3:1	4:1	5:1	40%
**Aqueous phase**	PBS	-					43%
**Size (d.nm)**		9.1 ± 0.1	9.4 ± 0.1	9.9 ± 0.1	10.29 ± 0.1	10.4 ± 0.3	
**PDI**		0.04 ± 0.1	0.03 ± 0.02	0.08 ± 0.007	0.05 ± 0.002	0.07 ± 0.01	
**Zeta potential (mV)**		−2.3 ± 0.2	−3.8 ± 1.1	−1.1 ± 0.2	−2.8 ± 0.4	−2.7 ± 0.4	
**Drug content (%)**		91.1 ± 2.1	94.9 ± 2.5	99.3 ± 1.3	96 ± 3.1	90.6 ± 3.2	

**Table 2:** Plasma pharmacokinetic parameters of IVMT-Rx-3 following oral administration of IVMT-Rx-3 dispersion and IVMT-Rx-3-ME2 to rats (mean ± SEM, n = 4). AUC_inf_, area under the curve from time zero to infinity; AUC_0→t_, area under the curve from time zero to the last sampling time point; C_max_, maximum observed concentration; t_1/2_, half-life.

Test Variables	IVMT-Rx-3 Dispersion	IVMT-Rx-3-ME2
Oral dose (mg/kg)	20	20
Tmax (h)	-	0.25 ± 0.00
T1/2 (h)	-	3.2 ± 0.98
Cmax (ng/mL)	-	170.5 ± 79.9
AUClast (h*ng/mL)	-	526.4 ± 461.0
AUCinf (h*ng/mL)	-	573.5 ± 492.8

## Data Availability

The original contributions presented in this study are included in the article. Further inquiries can be directed to the corresponding authors.
